# Prenatal Metal Exposure Alters the Placental Proteome in a Sex-Dependent Manner in Extremely Low Gestational Age Newborns: Links to Gestational Age

**DOI:** 10.3390/ijms241914977

**Published:** 2023-10-07

**Authors:** Anastasia N. Freedman, Kyle Roell, Eiona Engwall, Catherine Bulka, Karl C. K. Kuban, Laura Herring, Christina A. Mills, Patrick J. Parsons, Aubrey Galusha, Thomas Michael O’Shea, Rebecca C. Fry

**Affiliations:** 1 Department of Environmental Sciences and Engineering, Gillings School of Global Public Health, The University of North Carolina, Chapel Hill, NC 27599, USA; stasfree@live.unc.edu (A.N.F.); eieiona@email.unc.edu (E.E.); 2 Institute for Environmental Health Solutions, Gillings School of Global Public Health, The University of North Carolina, Chapel Hill, NC 27599, USA; kyle.roell@unc.edu; 3 College of Public Health, University of South Florida, Tampa, FL 33612, USA; bulka@usf.edu; 4 Department of Pediatrics, Division of Child Neurology, Boston Medical Center, Boston, MA 02118, USA; karl.kuban@bmc.org; 5 UNC Proteomics Core Facility, Department of Pharmacology, University of North Carolina at Chapel Hill, Chapel Hill, NC 27599, USA; laura_herring@med.unc.edu (L.H.); allie.mills@med.unc.edu (C.A.M.); 6 Wadsworth Center, New York State Department of Health, Albany, NY 12201, USA; patrick.parsons@health.ny.gov (P.J.P.); aubrey.galusha@health.ny.gov (A.G.); 7 Department of Environmental Health Sciences, School of Public Health, University of Albany, Rensselaer, NY 12222, USA; 8 Department of Pediatrics, School of Medicine, University of North Carolina, Chapel Hill, NC 27599, USA; moshea52@email.unc.edu; 9 Curriculum in Toxicology & Environmental Medicine, School of Medicine, University of North Carolina, Chapel Hill, NC 27599, USA

**Keywords:** metals, placenta, umbilical cord tissue, proteomics, preterm birth, sexual dimorphism

## Abstract

Prenatal exposure to toxic metals is associated with altered placental function and adverse infant and child health outcomes. Adverse outcomes include those that are observed at the time of birth, such as low birthweight, as well as those that arise later in life, such as neurological impairment. It is often the case that these adverse outcomes show sex-specific responses in relation to toxicant exposures. While the precise molecular mechanisms linking in utero toxic metal exposures with later-in-life health are unknown, placental inflammation is posited to play a critical role. Here, we sought to understand whether in utero metal exposure is associated with alterations in the expression of the placental proteome by identifying metal associated proteins (MAPs). Within the Extremely Low Gestational Age Newborns (ELGAN) cohort (*n* = 230), placental and umbilical cord tissue samples were collected at birth. Arsenic (As), cadmium (Cd), lead (Pb), selenium (Se), and manganese (Mn) concentrations were measured in umbilical cord tissue samples via ICP-MS/MS. Protein expression was examined in placental samples using an LC-MS/MS-based, global, untargeted proteomics analysis measuring more than 3400 proteins. MAPs were then evaluated for associations with pregnancy and neonatal outcomes, including placental weight and gestational age. We hypothesized that metal levels would be positively associated with the altered expression of inflammation/immune-associated pathways and that sex-specific patterns of metal-associated placental protein expression would be observed. Sex-specific analyses identified 89 unique MAPs expressed in female placentas and 41 unique MAPs expressed in male placentas. Notably, many of the female-associated MAPs are known to be involved in immune-related processes, while the male-associated MAPs are associated with intracellular transport and cell localization. Further, several MAPs were significantly associated with gestational age in males and females and placental weight in males. These data highlight the linkage between prenatal metal exposure and an altered placental proteome, with implications for altering the trajectory of fetal development.

## 1. Introduction

Toxic metals are ubiquitous in the environment, posing potential risks through air, soil, drinking water, and food [1]. Several metals/metalloids are of significant concern for public health. For instance, arsenic (As) is a known toxicant contaminating drinking water around the globe and is a critical issue in unregulated private well water in the United States (US) [2,3]. Likewise, excess manganese (Mn) has been identified in private well drinking water [4] and is known to be associated with adverse health outcomes such as impaired neurocognition [5]. Lead (Pb) exposure has drastically decreased since the 1970s in the US, though it is still present in water as a result of old plumbing systems [6]. Cadmium (Cd) is a developmental toxicant and carcinogen that is present in cigarette smoke and certain foods (e.g., leafy vegetables, shellfish, rice) and is known to bioaccumulate in plants grown in contaminated soils [7,8]. Selenium is an essential dietary trace element, and individuals are generally exposed to low levels through food, water, and air [9].

Given the many sources of metals and the increased risk for adverse health issues, there is a pressing need to both inform the public and understand the health effects associated with prenatal metal exposure. Among those at significant risk for adverse health effects associated with toxic metal exposure are pregnant women and children [10,11,12]. The Developmental Origins of Health and Disease (DOHaD) hypothesis suggests that a pregnant individual’s exposure to environmental influences during critical periods of fetal development has significant health consequences [13]. These consequences may range from adverse pregnancy and neonatal outcomes, such as low birthweight, or long-lasting health outcomes in the child, including altered development of tissues and organs as well as increased susceptibility to disease such as chronic heart or metabolic diseases [14,15,16]. Despite considerable efforts to raise awareness of the harms of toxic metals, recent studies highlight that continued exposures to these hazardous metals persist [17]. More specifically, exposure to toxic metals such as Cd, Pb, or metalloids such as As has been associated with developmental toxicity [18,19,20]. In contrast, essential elements such as Mn and Se are important for ensuring healthy and normal pregnancy outcomes [21,22]. However, deficiencies in or excess exposures to essential elements have been associated with adverse effects such as adverse pregnancy outcomes, neurotoxicity, or intrauterine growth restriction (IUGR) [22,23,24,25].

Associations between prenatal metals and adverse pregnancy/child health outcomes are well established [12,26]. However, the role of the placenta in this relationship and underlying mechanisms remain to be fully elucidated. The placenta, an organ originating from the fetus, is critical for proper fetal development, mediating the exchange of nutrients, waste, and gas between the mother and the fetus [27]. The placenta can also serve as a barrier, protecting the developing fetus from many harmful substances [28]. Nevertheless, metals can disrupt placental and fetal development by accumulating within or crossing the placental barrier [29,30,31]. Disrupted placental function during pregnancy is associated with adverse pregnancy outcomes, including increased risk of preterm birth, hypertension, or diabetes, as well as adverse later-life outcomes in the offspring [32,33]. Importantly, sexually dimorphic responses exist with regard to environmental exposures, pregnancy complications, and later-life health outcomes [34,35,36]. Sex-specific placental functioning may be driving these changes, as previous studies have demonstrated sexually dimorphic patterns in placental messenger RNA, microRNA, and CpG methylation expression differences [37,38]. It is imperative to elucidate the sexual dimorphism that underlies the effects of prenatal metal exposure on placental function.

In this study, we examined whether prenatal metal exposure is associated with the altered expression of the placental proteome, whether the expression of these proteins differed by sex, and whether the proteins were associated with placental weight or gestational age. Our study used an untargeted proteomics analysis within a unique repository of placentas and cord tissue samples collected at birth from the Extremely Low Gestational Age Newborn (ELGAN) study. Extremely low gestational age is defined as being born before 28 weeks’ gestation. Results from this study point to potential biomarkers of fetal metal exposure in utero. Furthermore, these findings may be applicable to the DOHaD framework, enhancing the understanding of the later-life consequences in relation to prenatal toxicant exposure.

## 2. Results

### 2.1. Demographic Characteristics of Study Participants

General characteristics of the ELGAN study participants (*n* = 230) are described in Table 1. Of these subjects, 99 (43%) of the ELGAN cohort were female, and 131 (57%) were male. The average maternal age was 29.6 years. Regarding insurance, 78 (33.9%) mothers had public health insurance during pregnancy. The mother’s educational status varied, with 93 (40.9%) participants receiving 12 or less years of education, whereas 49 (21.3%) participants received 13–15 years, and 84 (36.5%) received 16+ years of education. The majority of mothers (126 or 54.8%) were married and reported not smoking during pregnancy (206 or 89.6%). Regarding the fetal parameters, the average birthweight was 825 g, and the average weight of the placentas was 246 g. Additionally, the majority of deliveries occurred via C-section (162, 70.4%). Notably, none of the variables differ between males and females in Table 1 (*p*-value > 0.05). A table outlining whether trace element levels differed by various social determinants of health (e.g., maternal education, public insurance, eligibility for the Supplemental Nutrition Assistance Program (SNAP), and maternal socioeconomic score (SES) score) is available in Supplemental Table S1. Surprisingly, several toxic metals displayed higher levels among individuals with higher socioeconomic status.

### 2.2. Trace Elements in Umbilical Cord Tissue

Table 2 describes the levels of the five metals detected in cord tissue. Note that umbilical cord tissue was used in this study for the detection of metals, and placental tissue was used for the proteomics analysis, as these tissues are reflective of in utero fetal exposures. Maternal blood or serum were not available for analysis within this cohort. The metals were analyzed and then stratified by female and male umbilical cord tissue samples. Concentrations of trace elements in cord tissue across male and female specimens did not differ for all elements evaluated (*p*-value > 0.05). For trace element measurements among females, Se had the highest median concentration (0.880 µg/g), followed by Mn (0.342 µg/g), Pb (0.014 µg/g), As (0.004 µg/g), and Cd (0.001 µg/g). For trace element measurements among males, Se had the highest median concentrations (0.851 µg/g) in male umbilical cord tissue, followed by Mn (0.344 µg/g), Pb (0.017 µg/g), As (0.005 µg/g), and Cd (0.001 µg/g). Thus, within the female- and male-derived cord tissues, the order of the metals was selenium > manganese > lead > arsenic > cadmium. Correlation analysis identified that several of the elements were co-occurring in the cord tissue (Supplemental Table S2). Specifically, Pb and Cd were correlated (*r* = 0.416, *p* < 0.0001), Cd and As were correlated (*r* = 0.313, *p* < 0.0001), Pb and As were correlated (*r* = 0.277, *p* < 0.0001), and Se and Mn were correlated (*r* = 0.275, *p* < 0.0001).

### 2.3. The Identification of Metal-Associated Proteins

**Figure 1:**
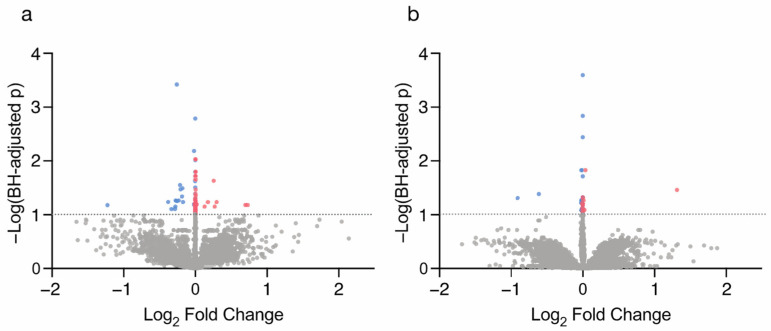
Volcano plots comparing the log2 fold changes versus *p*-values of the MAPs across (**a**) female and (**b**) male placentas. Proteins with negative fold changes (BH-adj *p*-value < 0.1) are indicated in blue. Proteins with positive fold changes (BH-adj *p*-value < 0.1) are indicated in red.

To identify metal-associated proteins (MAPs) that display altered expression in relation to umbilical cord metals, regression analyses were performed. Specifically, each of the metals was analyzed individually in relation to the expression of the 3454 proteins yielded from the proteomics analysis, controlling for maternal BMI category, SES score, and smoking status. This resulted in a β-estimate that indicates that for every 1 µg/g increase in a metal level in umbilical cord, there is a unit change in the placental protein expression. When analyzed among female placentas, a total of 90 protein–metal pairs were identified (BH-adj *p*-value < 0.1). When analyzed among male placentas, a total of 42 protein–metal pairs were identified (BH-adj *p*-value < 0.1). The fold change of all proteins and their expression levels in relation to metal levels was calculated and is presented in Figure 1.

**Figure 2:**
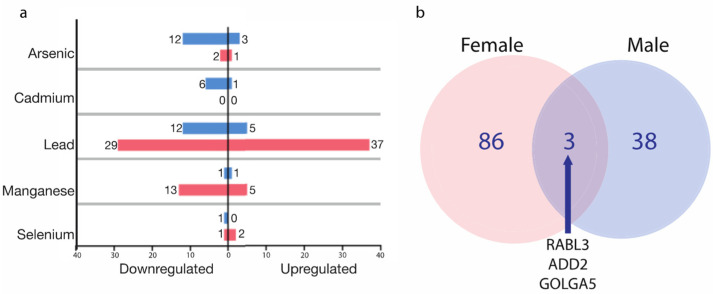
(**a**) Bar chart of number of MAPs and expression direction in female (red) and male (blue) placentas (BH-adj *p*-value < 0.1). (**b**) Venn diagram comparing male- and female-specific MAPs.

Of the 90 female-associated MAPs, 45 (50%) proteins displayed decreased expression, and 45 (50%) displayed increased expression (Figure 2a). Among females, Pb displayed the largest number of MAPs (*n* = 66). This was followed by Mn (*n* = 18 MAPs), As (*n* = 3 MAPs), and Se (*n* = 3 MAPs). There were no significant MAPs identified in relation to Cd, for females (Figure 2a, Supplemental Table S3). There was a single protein overlapping between As and Mn, specifically Cytochrome b-c1 complex subunit 9 (UQCR10). When considering this overlapping protein, a total of 89 unique female-associated MAPs were identified.

Of the 42 male-associated MAPs, 32 (76.2%) proteins demonstrated decreased expression, and 10 (23.8%) demonstrated increased expression (Figure 2a). Among males, Pb displayed the largest number of MAPs (*n* = 17). This was followed by As (*n* = 15 MAPs), Cd (*n* = 7 MAPs), Mn (*n* = 2 MAPs), and Se (*n* = 1 MAP). There was a single protein, F-box-like/WD repeat-containing protein TBL1XR1 (TBL1XR1), overlapping between Cd and Pb. When considering this overlapping protein, a total of 41 male-associated MAPs were identified (Figure 2, Supplemental Table S2).

A comparison of the 89 female-associated MAPs and 41 male-associated MAPs highlighted three overlapping proteins (Figure 2, Supplemental Table S3). Rab-like protein 3 (RABL3) was associated with Mn in females and Cd in males, Beta-adducin (ADD2) was associated with Mn in females and Pb in males, and Golgin subfamily A member 5 (GOLGA5) was associated with Mn in both females and males (Supplemental Table S3).

In a secondary non-stratified analysis (e.g., controlling for infant sex as a covariate), 23 protein–metal pairs were found to be significant as detailed in Supplemental Table S4. This resulted in 22 MAPs, as there was one protein (RABL3) that was significantly expressed in relation to two metals (Cd and Mn). In the non-stratified analysis, 17 (73.9%) proteins demonstrated decreased expression, and 6 (26.1%) demonstrated increased expression (Supplemental Figure S1).

Interestingly, while the essential metal Se demonstrated the highest concentrations across male and female placentas, Pb had the greatest number of associated MAPs (66 female MAPs, 17 male MAPs), and Se had the lowest number of associated MAPs (3 female MAPs, 1 male MAP). Finally, in this study, there were *n* = 46 twins from *n* = 23 unique mothers. A sensitivity analysis was performed to understand how the exclusion of one twin, chosen at random, from each pair would affect the expression of the MAPs. In the female-specific analysis, 78 out of 90 (81%) protein–metal pairs remain significant, and in the male-specific analysis, 34 out of 42 (81%) protein–metal pairs remain significant (BH-adj *p*-value < 0.1) (Supplemental Table S3). Sensitivity analysis results also highlight 17 out of 23 (74%) protein–metal pairs that remain significant in the non-stratified analysis (BH-adj *p*-value < 0.1) (Supplemental Table S4). This difference in protein expression is likely a result of the diminished sample size, rather than differences in biological responses to the metals.

### 2.4. Biological Functional Analysis of the MAPs

**Figure 3:**
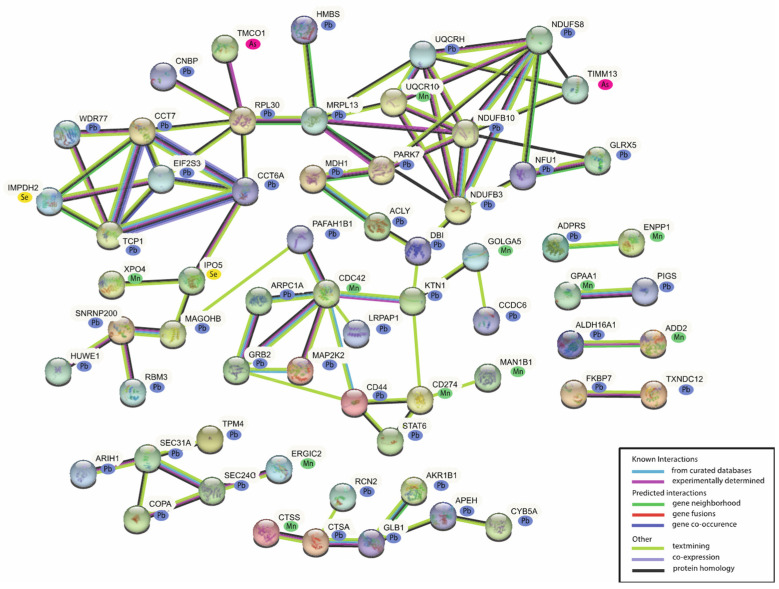
Network analysis of the female-associated MAPs. A total of 63 of the 89 MAPs have known protein–protein interactions.

Network and pathway enrichment analysis was conducted on the female- and male-associated MAPs to elucidate enriched biological processes among the proteins. In the female-associated network (*n* = 89 MAPs), 63 proteins demonstrated known protein–protein interactions. Within this network, the majority of the proteins with differential expression were associated with Pb (*n* = 48), followed by Mn (*n* = 11), As (*n* = 2), and Se (*n* = 2) (Figure 3).

The Reactome database was used for an in-depth evaluation of the functional enrichments within the MAPs. There were 74 significant pathways identified from the female MAPs (*p*-value < 0.05) (Supplemental Table S5). Among the female-associated MAPs, the top pathways identified are immune-system-related pathways, specifically the immune system (21 associated MAPs), innate immune system (15 associated MAPs), and adaptive immune system (10 associated MAPs). Proteins implicated across the various immune system pathways include Signal transducer and activator of transcription 6 (STAT6) (Pb; β = −0.002, *p*-value = 0.092), Cathepsin S (CTSS) (Mn; β = 0.256, *p*-value = 0.023), Programmed cell death 1 ligand 1 (CD274) (Mn; β = −0.283, *p*-value = 0.079), and E3 ubiquitin-protein ligase ARIH1 (ARIH1) (Pb; β = 0.002, *p*-value = 0.055) (Supplemental Table S4). Other notable pathways among the female MAPs include several relating to Rho GTPases, implicating the involvement of Cell division control protein 42 homolog (CDC42) (Mn; β = 0.129, *p*-value = 0.071) and Actin Related Protein 2/3 Complex Subunit 1A (ARPC1A) (Pb; β = −0.002, *p*-value = 0.019), among other proteins (Supplemental Table S5).

**Figure 4:**
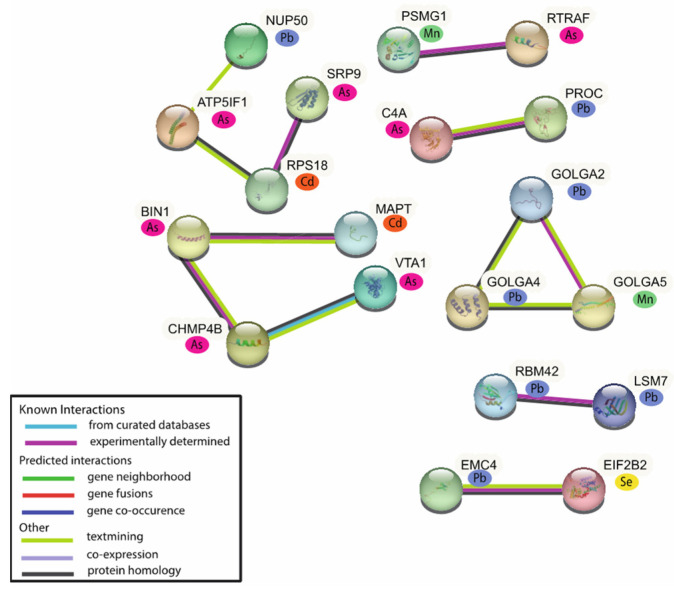
Network analysis of the male-associated MAPs. A total of 19 of the 41 MAPs have known protein–protein interactions.

For the network analysis of the male-associated MAPs (*n* = 41), 19 MAPs were included in the network. The majority of these MAPs are expressed in relation to As (*n* = 7) and Pb (*n* = 7), followed by Cd (*n* = 2), Mn (*n* = 2), and Se (*n* = 1) (Figure 4). The Reactome database identified 32 pathways from the male MAPs (*p*-value < 0.05) (Supplemental Table S5). The top pathways identified from the male MAPs include membrane trafficking (six associated MAPs), vesicle-mediated transport (six associated MAPs), and metabolism of RNA (six associated MAPs). Common proteins implicated across these top pathways include several golgin proteins, including Golgin A2 (GOLGA2) (Pb; β = −0.001, *p*-value = 0.048), Golgin A4 (GOLGA4) (Pb; β = −0.001, *p*-value = 0.099), and Golgin A5 (Mn; β = −0.615, *p*-value = 0.041). Other proteins highlighted in these pathways include Bridging Integrator-1 (BIN1) (As, β = −0.014, *p*-value = 0.084), and Vacuolar protein sorting-associated protein (VTA1) (As, β = −0.013, *p*-value = 0.082) (Supplemental Table S5).

### 2.5. Upstream Regulator Analysis of the MAPs

An analysis was performed to determine the transcriptional regulators of the sex-specific MAPs. Among the female MAPs (*n* = 89), there were 24 transcriptional regulators identified (*p*-value < 0.05) (Supplemental Table S6). Several significant upstream regulators target multiple molecules. Specifically, Distal-less homeobox 1 (DLX1) is the most significant transcription factor, targeting three proteins. Microphthalmia-associated transcription factor (MITF) is another significant transcription regulator that targets three proteins. GLI Family Zinc Finger 2 (GLI2) is a transcriptional regulator that targets genes encoding five proteins, all of which are Pb-associated female MAPs. There are also several proteins that are targeted by numerous transcription factors. For instance, CD274 is a predicted target of 10 different transcription regulators, and CD44 antigen (CD44) is a target of 11 different regulators (Supplemental Table S6).

Upstream regulator analysis of the male MAPs (*n* = 41) identified four transcriptional regulators (*p*-value < 0.05) (Supplemental Table S6). The most significant transcription factors are Myelin transcription factor 1 (MYT1) and Serine and arginine rich splicing factor 2 (SRSF2), which targets the Cd-associated MAP Microtubule-associated protein tau, (MAPT). The next most significant transcription factors are EHMT1 and E2F4, which each target two different MAPs (Supplemental Table S6).

### 2.6. Associations of MAPs with Placental Weight and Gestational Age

A secondary analysis was run on the sex-specific MAPs to assess whether they were associated with placental weight or gestational age. To assess this, correlation analyses were conducted on each of the MAPs in relation to placental weight and gestational age. Supplemental Table S7 illustrates the results of these correlation analyses. To detail, there were 11 MAPs (6 among females, 5 among males) significantly correlated with gestational age. Notable proteins include F-box only protein 50 (NCCRP1) (Pb; corr = 0.328, *p*-value = 0.039), which was positively correlated with gestational age, and Immunoglobulin lambda variable 3-25 (IGLV3-25) (Mn; corr = −0.292, *p*-value = 0.049), which was negatively correlated with gestational age in female placentas (Supplemental Table S7). Among male placentas, a notable protein included Melanoma-associated antigen D2 (MAGED2) (Cd; corr = −0.260, *p*-value = 0.003), which was negatively correlated with gestational age. Additionally, Immunoglobulin heavy constant gamma 4 (IGHG4) (As; corr = 0.200, *p*-value = 0.026), was negatively correlated with placental weight (Supplemental Table S7).

## 3. Discussion

Prenatal exposure to metals is associated with adverse pregnancy outcomes and later-life outcomes [39]. The placenta is posited to play an essential role in these relationships and thus presents as an important organ of study to elucidate the molecular mechanisms that underlie the effects of metals on adverse pregnancy and later-life outcomes [26]. Here, we evaluated placental protein expression in relation to prenatal metal levels in children born preterm from the ELGAN cohort and identified three major findings. First, the results support the a priori hypothesis that MAPs are enriched in pathways relating to inflammation and immune function, but it is also noteworthy that many of the identified proteins also highlight roles in Rho GTPase signaling and intracellular transport, enriched among female and male placentas, respectively. Second, while the metal levels in umbilical cord tissue were similar between females and males, sex-specific differences in protein expression in relation to metal levels are evident. Notably, the female MAPs were enriched for immune system, while male MAPs highlight roles in intracellular transport. Third, metal concentration in cord tissue does not correlate with the number of MAPs for a given metal. Finally, MAPs were associated with both placental weight and gestational age. Taken together, our results highlight the relationship between metal exposure during pregnancy and altered expression of the placental proteome, particularly regarding proteins involved in immune-related processes, in females.

This study is among the first to assess the placental proteome in relation to metals in utero in a cross-sectional analysis. Of 3454 measured proteins, we identified 89 MAPs in the female-specific analysis and 41 MAPs in the male-specific analysis. Several of the female-associated MAPs are enriched for their role in immune-related pathways, including CTSS, which was upregulated in relation to Mn. CTSS is essential for the proliferation of B cells in physiological humoral immune response and regulates antigen processing and T-cell-mediated immune responses [40]. ARIH1, which demonstrated increased expression in relation to Pb in females, has been shown to promote expression of proinflammatory cytokines [41]. Further, CD274, which is upregulated in relation to Mn levels, encodes an immune inhibitory receptor ligand and is expressed on activated T cells, B cells, and NKT cells to inactivate T cells or other immune cells, which is essential to the maintaining homeostasis of the immune system [42,43]. Notably, STAT6 demonstrates decreased expression in relation to Pb and is critical for normal functioning of the immune system by regulating the balance of inflammatory immune responses [44]. These results support our hypothesis that metal-associated proteins are involved in inflammatory and immune response, namely in females.

A comparison of the sex-stratified results identified three proteins with expression changes conserved in both male and female placentas but interestingly these were expressed differently in relation to specific metals. RABL3, which demonstrated decreased expression in both males and females, is involved in signaling pathways that control cell growth, maturation, and death, as it is required for KRAS signaling and modulation of cell proliferation [45]. ADD2 plays a role in promoting the assembly of the spectrin–actin network, maintaining the stability and structure of the cell membrane and shape and is downregulated in females and upregulated in males [46]. GOLGA5 is involved in maintaining Golgi structure and participates in intra-Golgi retrograde transport and is downregulated in both males and females [47].

Pb was associated with the largest proteomic signature, across both male and female placentas. Interestingly, the median concentrations of Pb in cord tissue are among the lowest, below Se and Mn, highlighting the fact that the proteomic changes were not predicted by metal levels. The Pb-associated MAP signatures were also more greatly pronounced in females compared to males. In this study, 66 (74%) proteins in females and 17 (41%) proteins in males exhibited expression changes in relation to Pb. Similar to our results, the current literature also shows Pb as having robust effects on transcriptomic changes. Specifically, in a prior study by our research group investigating the effects of metal and metal mixture exposures on differential gene expression, Pb had the greatest genomic response, though the majority of the genes demonstrated decreased expression [48]. In the present study, most of the Pb-associated proteins demonstrated increased expression among females and decreased expression among males. These findings that Pb-associated changes are more pronounced in females are reinforced in the literature. Prior research has found that females may be more susceptible to the effects of Pb on birth outcomes (i.e., head circumference, birthweight, and birth length), compared to male infants [49]. Additionally, in the study performed by Signes-Pastor et al., associations between Pb presence and female birth outcomes demonstrated the greatest effects (inverse associations), though Pb did not have the greatest concentration among the metals analyzed [49]. Further, in a mouse-model study exposing mice to Pb to understand differential immunotoxic outcomes based on sex, Pb-induced immunotoxic effects were more pronounced in female mice [50]. Taken together, these findings indicate stronger associations between prenatal lead exposure and susceptibility to placental proteomic dysregulation in females compared to males.

This study provides novel information on the association between metal exposure, sex-specific proteomic alterations in the placenta, placental weight, and gestational age. Here, we examined proteins because of their central role in cellular functions. Many factors influence protein expression, including mRNA abundance, and numerous factors control mRNA abundance, such as epigenetic modification and transcription factor abundance. Previous research performed by this research group examined the associations between placental gene expression networks and metal exposure in this cohort and found several key gene networks enriched for pathways related to Nuclear Factor Kappa-B (a master regulator of the immune system) [48]. Here, we analyzed proteins for their known roles as targets to various transcription factors. Among female-specific MAPs, many of the significant upstream regulators target proteins that display differential expression in relation to Pb. Such significant transcription factors include DLX1, which functions as a transcriptional regulator of signals from multiple TGF-B superfamily members and can inhibit several cytokine signaling pathways [51]. Another transcription factor that is predicted to target the Pb-associated MAPs is GLI2, which is also an effector of the TGF-B signaling and regulates stem cell proliferation [52,53]. Also, there are specific female MAPs that are commonly targeted by different transcription factors, including CD274 and CD44, which are key proteins enriched in the immune and innate immune system pathways. The top upstream regulator among the male-specific MAPs is MYT1, which influences cell proliferation, differentiation, and myeline gene transcription [54].

Analyses were also conducted to determine whether there were associations between MAP expression, gestational age, and placental weight. Several MAPs were associated with gestational age. To elaborate, NCCRP1 displayed decreased expression in relation to Pb in female placentas and was positively correlated with gestational age. Interestingly, NCCRP1 plays a role in the positive regulation of cell proliferation and displays differential expression in individuals who experienced multiple miscarriages [55]. IGLV3-25 displayed increased expression in relation to Mn in female placentas and was positively correlated with gestational age. This protein is involved in humoral immunity and participates in antigen recognition [56]. MAGED2 displayed decreased expression in relation to Cd in male placentas and was negatively associated with gestational age. Interestingly, MAGED2 is a hotspot for X chromosome-linked intellectual disability [57]. IGHG4 displayed increased expression in relation to As in male placentas and was positively correlated with placental weight. This protein is involved in activating the immune response and enables antigen binding [58].

In terms of public health implications of this work, there are several measures that could be taken to reduce a prenatal exposure to metals. If a pregnant individual is concerned about toxic metal exposure, they can reach out to their clinician to ask about biomonitoring. If they are a private well owner, they can contact their local health department about metal testing in their water. Among the actions that can be taken to reduce prenatal exposures are developing community engagement practices through researchers, local health authorities, and federal entities. Such practices can increase awareness for the exposure sources and effects of environmental metal exposures. Biomonitoring programs may also be implemented in an effort to screen maternal metal concentrations during pregnancy, particularly for vulnerable populations. Previous biomonitoring programs have been successful in tracking maternal metal concentrations in blood over the course of gestation [59]. Such programs were also able to examine relationships between metal concentrations and maternal demographic factors and inform mothers of ways to reduce chemical exposure. In tandem with biomonitoring, public health campaigns may also be created to offer assistance to at-risk mothers who experience exceedances in metal exposures by offering tools to decrease metal presence in homes, such as through offering water filters. Importantly, a continued search for more interventions or improved intervention techniques is vital for improving the safety of pregnant individuals.

While this study is among the first to examine prenatal metal exposure as it relates to the placental proteome, it is not without limitations. First, the ELGAN cohort comprises children who were all born extremely preterm, at or before 28 weeks’ gestation. Though it is imperative to study this population given their suspected vulnerability to environmental insults, the results from this research may not be generalized to children born at term. Further, it is important to note that the findings from this study do not inform the etiology of preterm birth but rather allow us to understand how the events that occur before birth may influence children born extremely preterm. Second, while these data show protein expression changes associated with metal presence in umbilical cord tissue, generally the protein level changes are small. Still, the fact that numerous placental proteins within key biological pathways displayed common dysregulation supports their potential biological impact. Third, umbilical cord tissue is a unique, yet understudied, tissue for examining prenatal exposures to chemicals. It is important to note that other studies have assessed trace elements in umbilical cord tissue and that these levels are comparable to those identified here [60,61,62]. Further, in order for metals to be detected in cord tissue, they would have passed through the placental tissue, thus directly exposing the placenta. In terms of comparability of umbilical cord tissue metal concentrations with other biological matrices such as the placenta, limited data suggest that metals present in placental tissue generally demonstrate higher concentrations than cord tissue for Cd, Se, and Pb. No umbilical cord metal concentration data currently exist with regard to As and Mn [30,61]. Of the studies that currently exist, the metal levels observed among the ELGAN cohort are comparable to populations in other studies that assessed Pb, Cd, Se, and As [30,61,62]. In relation to the use of cord tissue in this study, while it is not possible to identify the precise timing or duration of exposure, for several of these elements it is likely that levels detected in cord tissue represent exposure that happened during pregnancy [63,64].

In summary, we evaluated whether metal levels in umbilical cord tissue were associated with changes in the placental proteome in children born preterm. Further, we investigated whether changes in the placental proteome displayed sexually dimorphic patterns of protein expression, and whether these proteomic changes were correlated with select placental and neonatal outcomes. Our results indicate strong evidence of sexually dimorphic placental protein expression patterns, whereby distinct differences in protein expression changes may be observed between male and female placentas. Further, we identified a strong association between Pb levels and alterations in the placental proteome of males and females. Functional enrichment analyses of these proteins indicate the activity of pathways involved in immune response and cell transport/signaling. The correlation analysis established associations between select sexually-dimorphic MAPs, placental weight, and gestational age. These data contribute toward our understanding of the molecular mechanisms that underlie the relationship between harmful exposures to environmental metals and adverse pregnancy and later-life outcomes. Future research should incorporate mixture-based models to understand the effects of metal mixtures on placental protein changes. In addition, research should investigate the associations between these metal-induced protein expression patterns and later-life health outcomes such as autism spectrum disorder or impaired neurocognition. Understanding the mechanisms relating metal-associated protein expression patterns and adverse health outcomes later in life can inform efforts to identify intervention-based and solution-oriented research aimed toward mitigating the adverse effects of prenatal metal exposure.

## 4. Materials and Methods

### 4.1. The ELGAN Cohort

**Figure 5:**
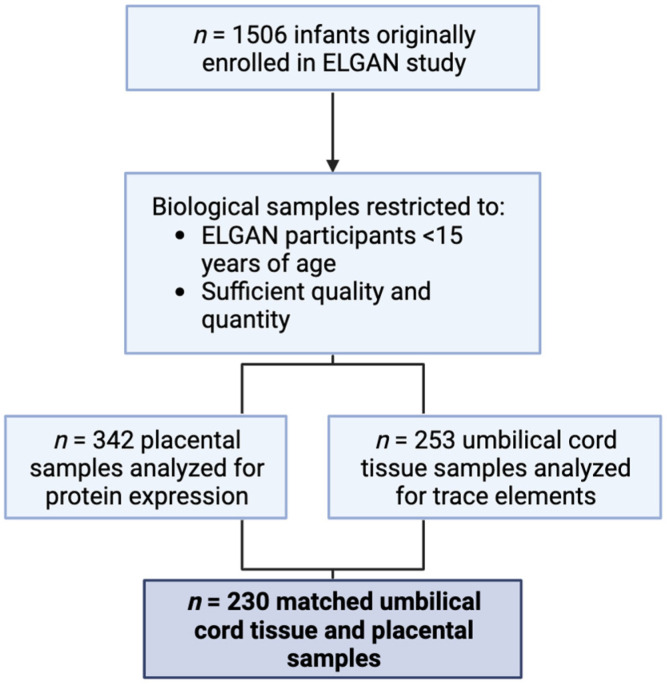
Diagram demonstrating the selection of the samples used for this study.

The Extremely Low Gestational Age Newborn (ELGAN) study is a prospective cohort study originally intended to understand structural and functional brain abnormalities in children born extremely preterm [65]. Extremely low gestational age is defined as birth before 28 completed weeks of gestation. From 2002 to 2004, infants born before 28 weeks’ gestation were enrolled. Enrollment occurred across 14 sites in five different states in the U.S. (Connecticut, Illinois, Massachusetts, Michigan, North Carolina). All mothers provided written informed consent, and Institutional Review Boards at each site approved the study procedures. Medical information was obtained from medical records, and a structured questionnaire collected self-reported information on maternal sociodemographic data, at enrollment. Umbilical cord tissue samples and placental samples were collected directly after delivery. Overall, 1506 infants were enrolled, accounting for multiple pregnancies. Selection of placental samples for sequencing was restricted to infants who survived to age 15 and samples that had sufficient quality and quantity for the proteomic sequencing (*n* = 342). Likewise, umbilical cord tissue samples were restricted to those that had sufficient quality and quantity for trace element analysis (*n* = 253). This yielded *n* = 230 matched placental-to-umbilical cord tissue samples that were used in analyses reported here (Figure 5).

### 4.2. Placental Tissue Collection and Quantification of Protein Expression

The ELGAN study included biobanks of two tissue types: (1) placenta and (2) umbilical cord tissue. Placental tissue samples were selectively used for omics-based analyses, with no remaining tissue available for trace element analysis. The methodology for their isolation is detailed below. Unfortunately, no maternal serum or neonatal cord blood was collected within the study. Methods for the placental tissue samples collected within the ELGAN cohort have been described elsewhere [37,66,67]. In brief, placentas were collected and biopsied at birth. A sample of about 1 g was removed from the base of the chorion. Samples were collected and stored in sterile 2 mL cryovials in Buffer RLT (Qiagen, Hilden, Germany) within a −80 °C freezer. For protein extraction, samples were thawed at 37 °C, and a sterile steel bead (Qiagen, Hilden, Germany) was added to each tube. Samples were then homogenized using a QIAGEN TissueLyser II (Qiagen, Hilden, Germany). A total of 300 µL of homogenate was used for downstream protein extraction. Four volumes (1.2 mL) of acetone (Thermo Fisher, Waltham, MA, USA) were added to each sample, followed by vortexing and centrifuging. A total of 1 mL of absolute ethanol (Thermo Fisher, Waltham, MA, USA) was added to each sample, followed by more vortexing and centrifuging. Protein pellets were dissolved in NaOH and sodium dodecyl sulfate (SDS) (Thermo Fisher, Waltham, MA, USA) then neutralized with HCl (Thermo Fisher, Waltham, MA, USA). Protease and phosphatase inhibitors were added (Thermo Fisher, Waltham, MA, USA). Protein content was then quantified using a Pierce BCA Protein Assay Kit according to manufacture protocol (Pierce, Waltham, MA, USA). For proteomics analysis, batch and sample numbers were randomly assigned. Approximately 50 µg of sample was reduced with 20 mM dithiothreitol (DTT) (Pierce, Waltham, MA, USA) for 10 min at 56 °C and alkylated with 40 mM iodoacetamide (Pierce, Waltham, MA, USA) for 30 min at room temperature. Samples were then loaded onto S-trap (Protifi, Fairport, NY, USA) columns according to the manufacturer’s recommended protocol. Samples were subjected to on-column digestion using trypsin (Promega, Madison, WI, USA) for 1 h at 47 °C at a 1:10 enzyme/protein ratio. Eluates were dried via vacuum centrifugation (Labconco, Kansas City, MO, USA), and peptide concentration was quantified using the Thermo fluorometric peptide assay. Samples were normalized to 0.3 µg/µL, and a pooled sample was created and analyzed alongside each batch. All samples were spiked with iRT standard peptides (Biognosys, Cambridge, MA, USA) prior to LC-MS/MS. Samples were then analyzed via LC-MS/MS using an Easy nLC 1200 coupled to a Fusion Lumos mass spectrometer (Thermo Fisher, Waltham, MA, USA). Samples were injected onto an Easy Spray PepMap C18 column (75 μm id × 25 cm, 2 μm particle size) (Thermo Fisher, Waltham, MA, USA) and separated over a 2 h method. The gradient consisted of 5–37% mobile phase B at a 250 nL/min flow rate, where mobile phase A was 0.1% formic acid (Pierce, Waltham, MA, USA) in water and mobile phase B was 0.1% formic acid in 80% acetonitrile (Sigma-Aldrich, St. Louis, MO, USA). The Lumos was operated in Data-Independent Acquisition (DIA) mode. A full MS scan (*m*/*z* 35–1200 *m*/*z*) was collected, and the resolution was set to 120,000 with a default charge state of 2, with a max injection time of 45 ms and automatic gain control (AGC) target of 250%. Following the full MS scan, the product ion scan was collected at a resolution of 30,000, with higher collision dissociation (HCD) set to 30, AGC target set to 2000%, maximum injection time set to 54 ms, and 31 *m*/*z* precursor isolation windows. Raw files were analyzed using Spectronaut v15 (Biognosys, Cambridge, MA, USA) against the reviewed human proteome database from Uniprot (UP000005640, downloaded October 2020 containing 20,381 sequences). The following settings were used: enzyme specificity set to trypsin, up to two missed cleavages allowed, cysteine carbamidomethylation set as a fixed modification, methionine oxidation and N-terminal acetylation set as variable modifications. Precision iRT calibration was enabled, and the profiling strategy was set to iRT profiling. A false discovery rate (FDR) of 1% was used to filter all data. Normalization and imputation were disabled in Spectronaut. Further downstream processing, including normalization, random-forest-based imputation, and batch correction, were performed using the vsn R package (v3.62.0) [68], the MissForest R package (v1.5) [69], and the ComBat function from the sva package (v3.42.0) [70], respectively, in R. Over 84,000 peptides were identified from roughly 5000 proteins, pre-filter. Downstream data filtering removed proteins with no quantification, over 50% missing values, and <33% q-values < 0.1, yielding 3454 proteins passing cutoffs.

### 4.3. Umbilical Cord Tissue Collection and Trace Element Measurements

As detailed above, the ELGAN biobank was limited to placenta and umbilical cord tissue. The scientific support for the appropriate use of umbilical cord includes established comparisons of metals between the cord and placenta [30,61]. Methods for umbilical cord tissue collection and processing have been described elsewhere [17]. Briefly, immediately after delivery, two 1 cm segments of the umbilical cord were collected with a stainless-steel scalpel blade using sterile techniques. Samples were then placed into separate cryostorage vials and into liquid nitrogen for transport to a −80 °C freezer before shipping to the Wadsworth Center for trace element determination. Upon arrival at the Wadsworth Center, samples were thawed and sectioned into roughly 0.4 g (wet mass; ~0.05 g dry mass) pieces using high-purity tantalum tools that were created in-house for use in trace element measurements. All samples were rinsed with double-deionized (DDI) (Thermo Scientific Barnstead, Waltham, MA, USA) water to remove superficial blood and placed in a clean 13 mL acid-washed tube. Samples were then freeze-dried to constant mass using a slow 5-step program to ensure thorough removal of water content. Batches of samples were digested in concentrated, double-distilled HNO_3_ using a microwave-assisted reaction system (MARS 6, Matthews, NC, USA) with closed “Xpress” vessels and the “One-Touch Animal Tissue” method. For acid digestion, ACS Reagent grade HNO_3_ was double-distilled in-house using a DuoPUR Sub-boiling point distillation system (Milestone Inc, Shelton, CT). Digests were then diluted to roughly ~10 g with DDI water and stored at 4 °C pending analysis. An optimized instrumental method was used to analyze digestates, leveraging an Agilent 8900 ICP-MS/MS equipped with an SPS 4 autosampler and an Octopole Reaction System (ORS) with axial acceleration technology. Two cell modes were leveraged—He was used in Kinetic Energy Discrimination KED mode to reduce polyatomic interferences on ^78^Se, and O_2_ was used as a reaction gas for interferences affecting ^55^Mn and ^75^As and ^å206, 207, 208^Pb. In O_2_ gas mode, As was monitored as ^75^As^16^O, i.e., mass shifted to remove interferences; Mn and Pb were measured on mass. The method was validated against two Standard Reference Materials (SRMs) from the National Institute of Standards and Technology (NIST): NIST SRM 1577b Bovine Liver and NIST SRM 1577c Bovine Liver. NBS 1577 Bovine Liver and SRM 8414 Bovine Muscle, were the two additional SRMs used for additional quality control. The SRMs were freeze-dried, digested, and analyzed alongside the umbilical cord tissue samples. Additionally, values for sample spikes (within ±20% recovery), duplicates (within ±20%), blanks, and calibrators were monitored carefully throughout the study. Limits of detection (LODs) for the 5 metals analyzed were as follows: As (0.00042 µg/g), Cd (0.00032 µg/g), Mn (0.010 µg/g), Pb (0.0026 µg/g), and Se (0.10 µg/g). All samples had detectable concentrations of As, Mn, Pb, and Se, while 98% had detectable levels of Cd. Samples at or below the LOD were imputed as LOD/√(2), as is commonly implemented in environmental chemistry data [71,72]. All statistical analyses were conducted in R (v4.1.2) [73]. Descriptive statistics (median, mean, minimum, maximum, range) were calculated to describe the distribution of the metals. To test for normality in cord metals data, a Shapiro–Wilks test was used. As no metal was normally distributed, Spearman rank correlation was used to evaluate pairwise correlations between the metals.

### 4.4. Covariate Selection

For all models, covariates were selected a priori based on a directed acyclic graph approach [74]. Additionally, surrogate variables were added to account for sample heterogeneity. The previous literature supports that social determinants of health may confound the relationship between exposures and outcomes [75,76]. As such, these variables have been included in the model as covariates. Selected covariates included maternal pre-pregnancy BMI (underweight, normal, overweight, obese), maternal smoking during pregnancy (yes/no), and maternal socioeconomic status (SES) score. In detail, maternal SES score was calculated based on a summative count of single marital status, less than college education, public health insurance, and eligibility for the Supplemental Nutrition Assistance Program (SNAP) as detailed previously [77]. Infant sex (male/female) was included as a covariate in the non-sex-stratified analysis to ensure precision and due to the sexually dimorphic nature of the placenta. Data were missing for SES score (*n* = 7), maternal pre-pregnancy BMI (*n* = 7), and maternal smoking during pregnancy (*n* = 4). Random forest modeling was utilized to impute missing covariates using the missForest R package, prior to modeling (v.1.4) [69,78]. The missForest package employs a nonparametric imputation method that works for categorical and continuous data. Each variable was fit on a random forest model and then used to predict the missing data. These two steps are repeated iteratively to continuously update the imputed data, until the stopping criterion is met, which is when the differences between the previous imputation result and the new imputation result is increased [69]. The out-of-bag imputation error value for the normalized root mean squared error was 0.006, indicating good performance of the random forest imputation [69].

### 4.5. Significance Tests across Demographic Characteristics

In order to assess how the metal levels compare across various demographic characteristics, several tests of significance were conducted. Because the metals data are not normally distributed, non-parametric tests were conducted. These findings are reflected in Supplemental Table S1. For characteristics with two variables (i.e., insurance status and eligibility for the SNAP), Mann–Whitney–Wilcoxon tests were run for each trace element in this study. For variables with more than two variables (i.e., maternal education and maternal SES score), Kruskal–Wallis tests were conducted. Significance was set at *p*-value < 0.05. To test whether there was a significant difference between male and female infants across the demographic characteristics in Table 1, *t*-tests and chi-square tests were performed. For demographic characteristics that contained a singular variable with data represented as mean [SD], *t*-tests were conducted between males and females. These characteristics include maternal age, gestational age, birthweight, and birthweight z-score. For demographic characteristics with multiple categories within a variable and data represented as *n* (%), chi-square tests were run. These variables include mode of delivery, duration of labor, mother’s education, race, marital status, pre-pregnancy BMI, smoking status, and SES score.

### 4.6. Evaluation of the Relationship between Trace Elements and Placental Protein Expression

To assess expression changes of individual proteins in relation to metals, robust linear regression models were fit between the protein expression values as the dependent variable to the metals concentration as the independent variable using Limma (v3.50.3) [79]. Models were adjusted for covariates as previously described. Moderated test statistics were calculated using the ebayes function within the Limma R package. This procedure shrinks the estimated sample variances toward a global trend, resulting in more stable inferences. Each of the five metals were separately analyzed against 3454 proteins from the proteomics assessment (post-filtering). The estimate from these regressions represents the expression changes in the protein per unit increase in the metal concentration. To address multiple testing, *p*-values were adjusted using the Benjamini and Hochberg (BH) procedure [80]. All statistical analyses were conducted in R (v4.1.2) [73]. A log2 fold change was also calculated for each MAP by dividing the average of the highly expressed (>median) proteomics values by the average of the less expressed (<median) proteomics values. Several of the matched placental and umbilical cord tissue samples used in this study came from twins. A sensitivity analysis was conducted, comparing the effects of twin inclusion and exclusion on expression of the metal-associated proteins. Within this analysis, one twin from each pair (*n* = 46 twins from *n* = 23 unique mothers) was randomly chosen from each set of multiples to be included in this analysis.

### 4.7. Assessment of MAP Associations with Pregnancy or Neonatal Outcomes

To assess whether any MAPs were associated with pregnancy or neonatal outcomes, correlation analyses were conducted. Pregnancy outcomes evaluated included gestational age and placental weight. Using the significant sex-stratified MAPs (*n* = 89 female and *n* = 41 male), Pearson correlations were run for each MAP in relation to either gestational age or placental weight. A BH-adjusted *p*-value was used to account for multiple testing. Significance was set at a BH-adjusted *p*-value of <0.1.

### 4.8. Network Interaction Analysis

To understand the biological functional implications of proteins differentially expressed in relation to metals, network enrichment analyses were performed using the Search Tool for the Retrieval of Interacting Genes/Proteins (STRING) [81]. All differentially expressed proteins with a significant association with a metal were used as input data, for the female- and male-associated proteins. Networks were constructed based on known protein–protein interactions and other molecular interactions.

### 4.9. Biological Functional Analysis

Functional annotations of the MAPs were assessed using Reactome [82], and upstream enrichment analysis was evaluated using Ingenuity Pathway Analysis (IPA, Ingenuity Systems, Redwood City, CA, USA). Reactome is an open-source manually curated pathway database providing bioinformatic tools for the interpretation and analysis of protein pathways. Enriched pathways from Reactome are defined as those with a *p*-value < 0.05. The female-associated MAPs (*n* = 89) and male-associated MAPs (*n* = 41) were uploaded into the interface, and the full analysis results were exported and filtered for significance (*p*-value < 0.05). To understand the mechanisms of protein regulation underlying the observed changes, the upstream regulator analysis in IPA was used to identify transcriptional regulators of the proteins. The upstream regulator analysis was restricted to only include transcriptional regulators that were significant at *p*-value < 0.05.

## Figures and Tables

**Table 1:** Sociodemographic characteristics of ELGAN sub-cohort analyzed in this study. Data included reflect *n* (%) or mean [SD]. *p*-values reflect statistical differences between males and females.

	All Participants (*n* = 230)	Females (*n* = 99)	Males (*n* = 131)	*p*-Value
**Maternal age (average)**	29.6 [6.70]	28.9 [6.59]	30.1 [6.75]	0.159
**Gestational age in weeks (average)**	26.0 [1.26]	26.1 [1.22]	25.9 [1.28]	0.230
**Birthweight in grams**	825 [183]	810 [188]	839 [179]	0.282
**Birthweight z-score**	−0.22 [1.03]	−0.37 [1.15]	−0.10 [0.91]	0.057
**Placental weight in grams**	246 [121]	260 [122]	236 [119]	0.139
**C-section delivery**				
Yes	162 (70.4%)	70 (70.7%)	92 (70.2%)	1.000
No	68 (29.6%)	29 (29.3%)	39 (29.8%)	
**Duration of labor**				
0 h	64 (27.8%)	28 (28.3%)	36 (27.5%)	0.851
≤12 h	50 (21.7%)	23 (23.2%)	27 (20.6%)	
>12 h	116 (50.4%)	48 (48.5%)	68 (51.9%)	
**Public insurance**				
No	150 (65.2%)	60 (60.6%)	90 (68.7%)	0.262
Yes	78 (33.9%)	38 (38.4%)	40 (30.5%)	
“Missing”	2 (0.87%)	1 (1.01%)	1 (0.76%)	
**Mother’s education (years)**				
≤12	93 (40.9%)	42 (42.4%)	51 (38.9%)	0.260
3–15	49 (21.3%)	24 (24.2%)	25 (19.1%)	
16+	84 (36.5%)	30 (30.3%)	54 (41.2%)	
“Missing”	4 (1.74%)	3 (3.03%)	1 (0.76%)	
**Mother’s race**				
White	136 (59.1%)	57 (57.6%)	79 (60.3%)	0.684
Black	70 (30.4%)	33 (33.3%)	37 (28.2%)	
Other	21 (9.13%)	8 (8.08%)	13 (9.92%)	
“Missing”	3 (1.30%)	1 (1.01%)	2 (1.53%)	
**Marital status (married)**				
No	104 (45.2%)	49 (49.5%)	55 (42.0%)	0.318
Yes	126 (54.8%)	50 (50.5%)	76 (58.0%)	
**Maternal pre-pregnancy BMI**				
Underweight	18 (7.83%)	6 (6.06%)	12 (9.16%)	0.573
Normal	115 (50.0%)	46 (46.5%)	69 (52.7%)	
Overweight	39 (17.0%)	19 (19.2%)	20 (15.3%)	
Obese	53 (23.0%)	25 (25.3%)	28 (21.4%)	
“Missing”	5 (2.17%)	3 (3.03%)	2 (1.53%)	
**Maternal smoking while pregnant**				
No	204 (88.7%)	86 (86.9%)	118 (90.1%)	0.765
Yes	23 (10.0%)	11 (11.1%)	12 (9.16%)	
“Missing”	3 (1.30%)	2 (2.02%)	1 (0.76%)	
**Maternal SES score**				
0	109 (47.4%)	43 (43.4%)	66 (50.4%)	0.149
1	36 (15.7%)	13 (13.1%)	23 (17.6%)	
2	55 (23.9%)	26 (26.3%)	29 (22.1%)	
3	23 (10.0%)	15 (15.2%)	8 (6.11%)	
4	7 (3.04%)	2 (2.02%)	5 (3.82%)	

**Table 2:** Concentrations of the five trace elements measured in umbilical cord tissue from females (*n* = 99) and males (*n* = 131). All values are in µg/g. *p*-values reflect statistical differences between males and females.

Metal	Sex	Median	Mean	Minimum	Maximum	*p*-Value
Selenium (Se)	Female	0.880	0.877	0.436	1.610	0.493
	Male	0.851	0.892	0.600	1.980	
Manganese (Mn)	Female	0.342	0.432	0.101	5.577	0.310
	Male	0.344	0.373	0.194	1.699	
Lead (Pb)	Female	0.014	0.029	0.003	0.350	0.463
	Male	0.017	0.036	0.003	0.893	
Arsenic (As)	Female	0.004	0.006	0.001	0.078	0.600
	Male	0.005	0.007	0.001	0.067	
Cadmium (Cd)	Female	0.001	0.005	0.0002	0.075	0.204
	Male	0.001	0.045	0.0002	4.06	

## Data Availability

All data generated or analyzed in this study are publicly available. The mass spectrometry proteomics data have been deposited to the ProteomicsXchange Consortium via the PRIDE partner repository with the dataset identifier PXD041694.

## Supplementary Materials

The following supporting information can be downloaded at: https://www.mdpi.com/article/10.3390/ijms241914977/s1.
