# Supplementary material for: Prenatal Metal Exposure Alters the Placental Proteome in a Sex-Dependent Manner in Extremely Low Gestational Age Newborns: Links to Gestational Age
Source: Int J Mol Sci. 2023 Oct 7;24(19):14977. doi: 10.3390/ijms241914977 (PMC10573797; doi:10.3390/ijms241914977)
Supplement: Supplementary file 1 [file ijms-24-14977-s001.zip › Figure S1.pdf]

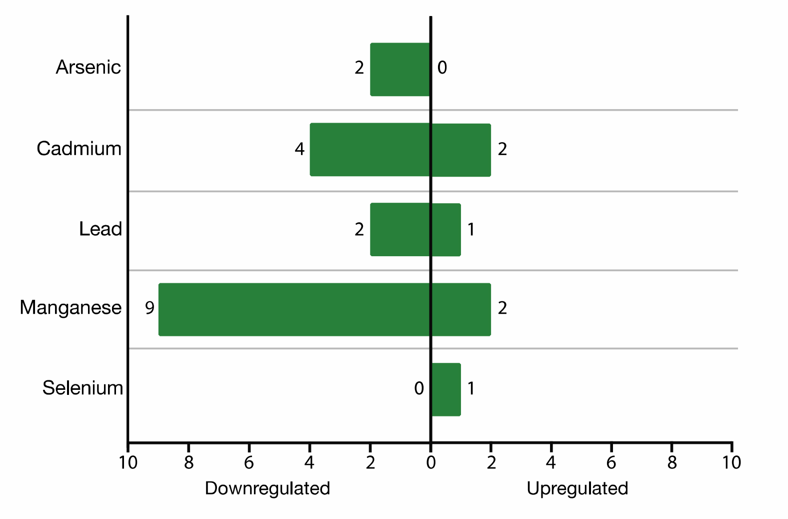

Figure S1. Bar chart of number of MAPs and expression direction in the non-sex-stratified analysis (BH-adj *p*-value < 0.1).
